# Undergraduate medical education in Nepal: one size fits all?

**DOI:** 10.3352/jeehp.2011.8.9

**Published:** 2011-10-01

**Authors:** P. Ravi Shankar

**Affiliations:** Department of Medical Education, KIST Medical College, Lalitpur, Nepal.

Nepal is a small developing country in South Asia situated between China and India. The last fifteen years have seen a tremendous growth in the number of medical schools in the country. At the beginning of August 2011 there were eighteen medical schools and all except four were in the private sector [1]. The private schools are run by both Nepalese and foreign (mainly Indian) groups. Nepalese-owned schools provide 10% and foreign owned schools 20% of the total seats with full tuition scholarship to students, who are selected through an entrance examination conducted by the Ministry of Education. These students have to serve in health facilities in rural Nepal for two years after graduation and are becoming an important source of support to Nepal's health system [2].

## DIVERSITY OF STUDENTS

Among government-run schools, the National Academy of Medical Sciences (NAMS) is a postgraduate training institution attached to Bir Hospital, the oldest hospital in Nepal. The Institute of Medicine (IOM) was the first medical school to be started in the late 1970s. IOM admits students from other countries who are paying privately to the undergraduate medical (MBBS) course. The other two schools, BP Koirala Institute of Health Sciences (BPKIHS) and Patan Academy of Health Sciences (PAHS) admit students paying partial and full tuition to the MBBS course. Colleges in Nepal also admit students from India, Sri Lanka, and from developed nations who pay higher tuition fees than Nepalese students. PAHS does not admit foreign students to the MBBS course.

In Nepal, there are scholarship students, Nepalese self-financing students, and self-financing students from South Asia and from other regions. Scholarship students have to serve in rural areas for two years after graduation. At PAHS all students (scholarship and self-financing) will be expected to serve in rural areas for a period of time according to a sliding scale (depending on the method of financing) after graduation. The author is conducting a study on student perceptions about working in rural Nepal after graduation from KIST Medical College. Preliminary results indicate certain self-financing students may be highly motivated to emigrate to developed nations, mainly the United States and Australia, after graduation. This is also the author's personal observation after teaching in self-financing schools in Nepal for about twelve years. Tuition fees for self-financing students are high, ranging from US$32,000-40,000 for Nepalese students, US$50,000-53,000 for Indian students, and US$60 000 for students from other countries [3]. Sri Lanka has only one private medical school and many Sri Lankan self-financing students are enrolled in Nepalese schools. Considering the high tuition and other fees students must pay, working in the government health sector or in rural Nepal does not give an adequate return on their investment.

## MEDICAL SCHOOL CURRICULA

The faculties in Nepalese medical schools mainly come from Nepal and India, with a few faculty members coming from other countries. Many faculties are not familiar with the sociocultural milieu of Nepal and with healthcare delivery in rural areas. The duration of the MBBS course is four and half years followed by a year of rotating internship which foreign students can do in their home countries. At PAHS, the MBBS course is of six years duration (with internship) and there is a premedical phase of 6 months where students review science subjects and English and are introduced to rural and underserved community experiences. The medical schools are affiliated with and follow the curriculum of two Nepalese universities, Tribhuvan University, a government university, and Kathmandu University, a private university. BPKIHS and PAHS are autonomous institutions. The curricula in most schools are broadly similar. During the first two years, students learn the basic science subjects of anatomy, physiology, biochemistry, pathology, microbiology, and pharmacology along with community medicine and early clinical exposure. The curriculum of all universities have the ostensible purpose of creating doctors for rural Nepal, though only a small percentage eventually work in rural areas. At PAHS the curriculum has been designed to admit and train a high percentage of students from rural Nepal and to model rural service and social responsibility throughout the medical course.

## STANDARD CURRICULUM FOR ALL STUDENTS

Medical education and medical schools especially in South Asia have long espoused the principle that one 'size' or 'style' of medical education fits all. With the rapid advancements occurring in medical knowledge, it may be time to recognize that no medical student can learn all 'necessary' facts and skills. The knowledge and skills to be taught and learnt may be determined by students' career plans after graduation and where he or she plans to work. In Nepal and in many other countries, self-financing students are supported by their families or educational loans and their primary motive may be to recover the high investment as quickly as possible after graduation. The government does not provide scholarship to these students and many students may not consider having a 'social' and 'community' obligation after graduation. Working and settling in developed nations or in specialty and super-specialty hospitals in urban areas in the home country is their priority. However, there may be indirect investments by the country/community in terms of faculty dedication, faculty professional development, and other inputs devoted to the education of these students. Ideally all medical students should feel an obligation towards their country and serving the underserved population regardless of whether the school pays their tuition fees or not. Private medical colleges also have students who have to work in rural areas after graduation. In many cases, these two groups of students i.e., students who are required to work in rural areas and those who are not required, have a different outlook and way of life. Scholarship students can become 'corrupted' by the ostentatious lifestyle of a few self-financing students, look down on rural service, and develop the desire to make money quickly. A self-financing seat costs about 3 million Nepalese rupees and the overall expenses at the end of the course may be around 3.5 to 4 million. Because of this, many self-financing students have to be from a strong financial background. In Nepal, government student loans are absent, and banks may require collateral and good financial status to approve loans. However, it is also possible that students from urban areas come to know and appreciate the needs of their less fortunate countrymen by working and studying alongside students from rural backgrounds.

## TRAINING FOR WORKING IN RURAL NEPAL

The training does not prepare scholarship students for their service in rural Nepal. Though students spent time in communities, the training is not adequate to enable them to work independently in rural areas, handle administrative responsibilities, interact with rural communities, and live in rural Nepal. PAHS has carefully designed its curriculum to prepare students for practice in rural Nepal and it would be interesting to follow the progress of this 'different' medical school and its students. Many self-financing students have their goals set firmly on examinations like the United States Medical Licensing Examination and postgraduate entrance examinations and/or establishing an urban practice. Teachers suffer from an inadequate knowledge of conditions in rural Nepal and of the government healthcare system. Many have not spent adequate time in rural areas. A similar situation may exist in medical schools in other south Asian countries and other developing nations. Privatization of medical education and admission of scholarship students to these schools has created a student body with diverse educational needs and outlooks. Foreign students further add to the diversity. Will this diverse group of students require curricula and teaching methods tailored to their specific needs? One approach could be of a core curriculum for all students and of electives which students can pursue according to their interests. This concept has not yet gained acceptance in Nepal. Another approach could be to design a medical school like PAHS with a common curriculum of social responsibility for all its students but with electives that students can pursue according to their interests and career choices. In this scenario it may be time to review and reconsider the old basic principle of medical schools in Nepal and other countries that states, 'One size of medical education fits all students'.
